# Supplementary material for: Activity enhancement of cobalt catalysts by tuning metal-support interactions
Source: Nat Commun. 2018 Oct 26;9:4459. doi: 10.1038/s41467-018-06903-w (PMC6203836; doi:10.1038/s41467-018-06903-w)
Supplement: Supplementary file 1 — Supplementary Information [file 41467_2018_6903_MOESM1_ESM.pdf]

Supplementary Information

Activity enhancement of cobalt catalysts by tuning metal-support interactions

Hernández Mejía *et al.*

Contents

Supplementary Figures 2
Supplementary Tables 9
Supplementary Methods 13
Supplementary References 14

Supplementary Figures

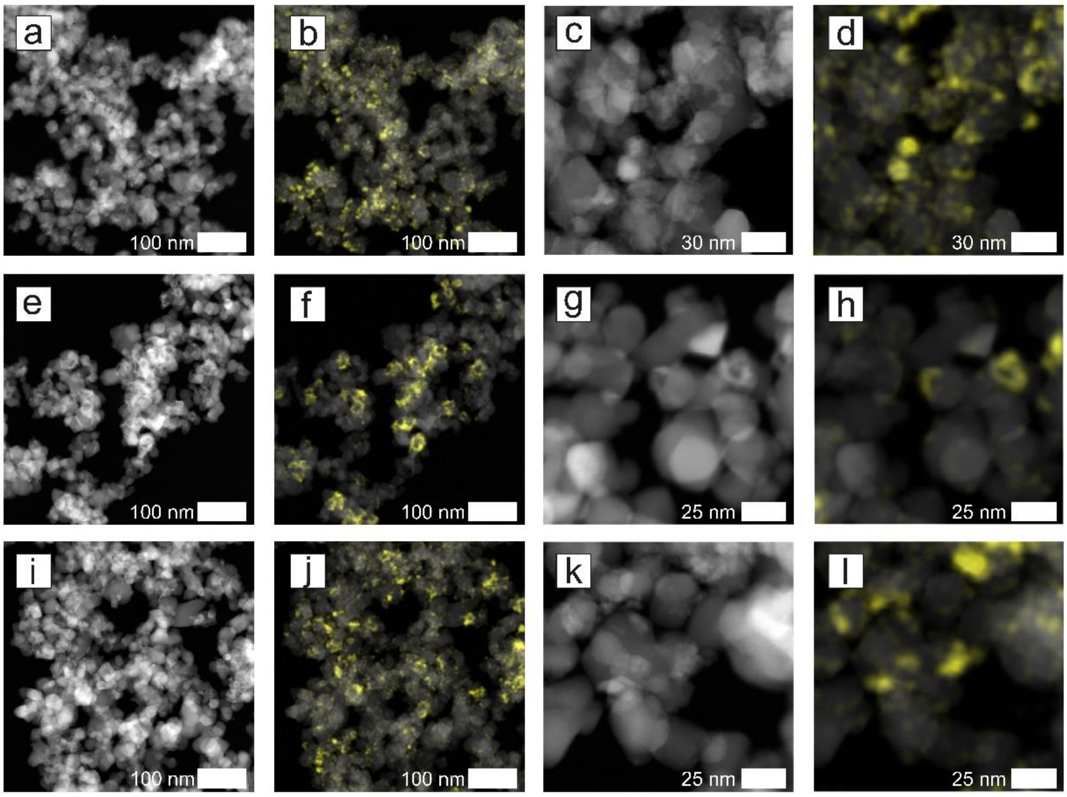

Supplementary Figure 1. Additional electron microscopy images of the TiO_2-supported samples upon reduction-oxidation-reduction treatments. STEM-HAADF combined with EDX mapping of Co/TiO_2 at various stages of the ROR treatment. Co is depicted in yellow. **a, b, c, d,** Samples after reduction at 350 °C and passivation (R-TiO_2); **e, f, g, h,** samples after reduction at 350 °C and oxidation at 200 °C (RO200-TiO_2); **i, j, k, l,** samples after reduction at 350 °C, oxidation at 200 °C and reduction at 220 °C (RO200R-TiO_2) and passivation.

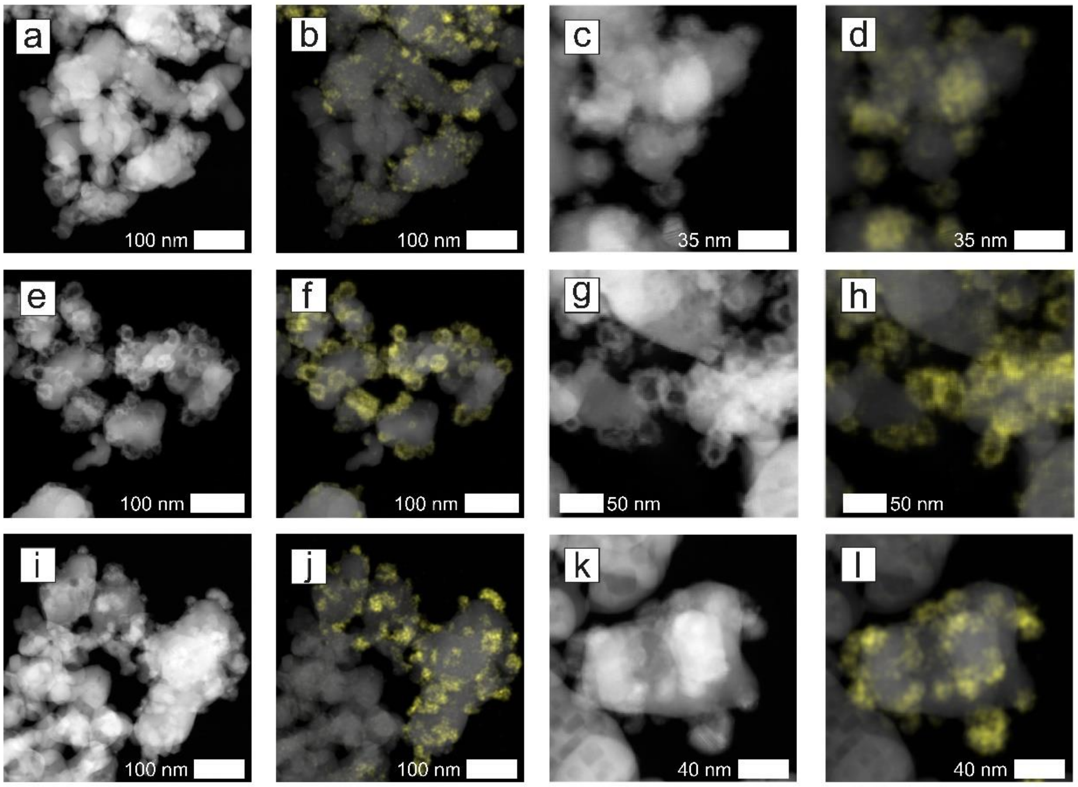

Supplementary Figure 2. Additional electron microscopy images of the Nb_2O_5-supported samples upon reduction-oxidation-reduction treatments. STEM-HAADF combined with EDX mapping of Co/Nb_2O_5 at various stages of the ROR treatment. Co is depicted in yellow. **a, b, c, d,** Samples after reduction at 350 °C and passivation (R-Nb_2O_5); **e, f, g, h,** samples after reduction at 350 °C and oxidation at 200 °C (RO200-Nb_2O_5); **i, j, k, l,** samples after reduction at 350 °C, oxidation at 200 °C and reduction at 220 °C (RO200R-Nb_2O_5) and passivation.

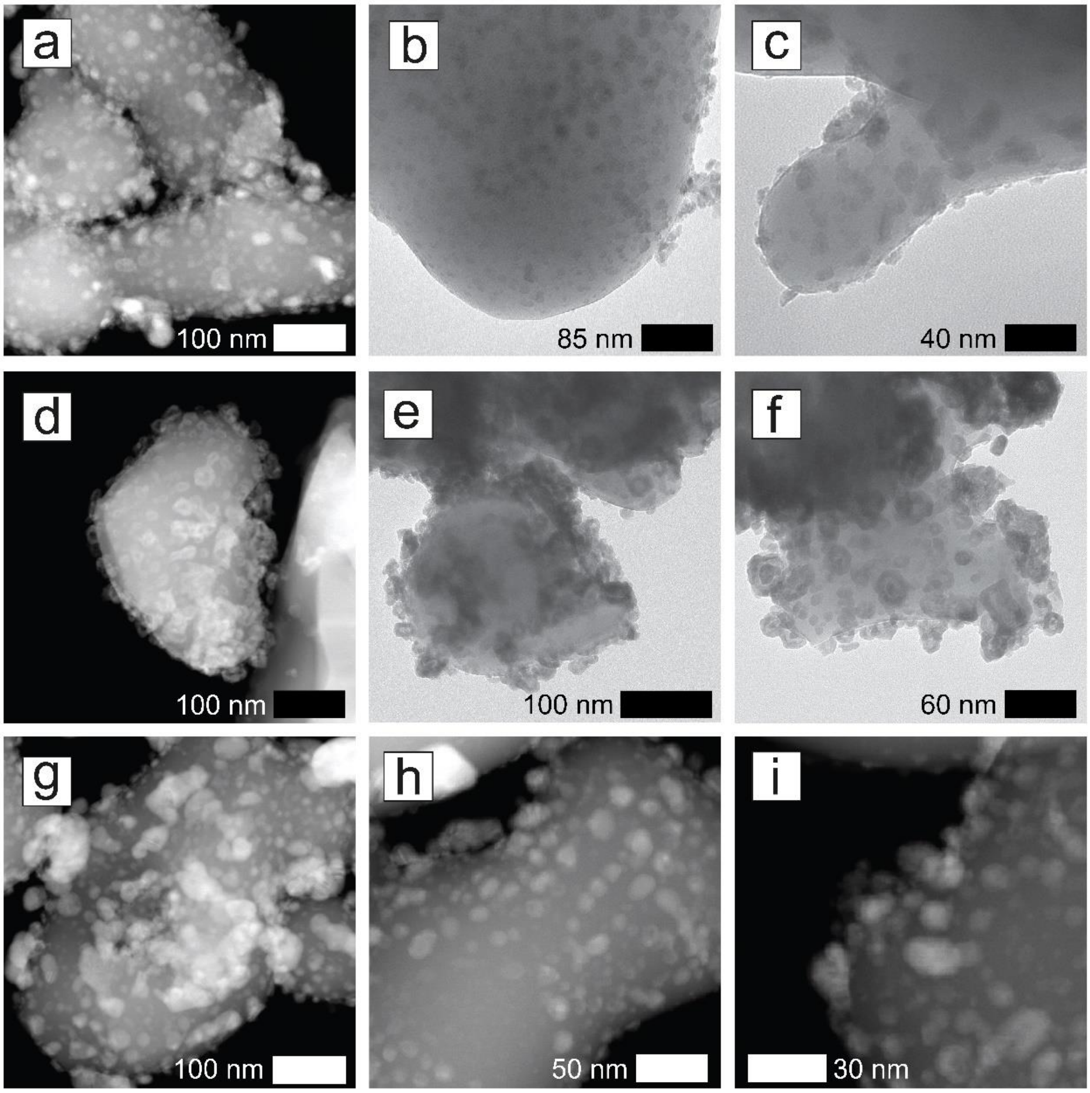

Supplementary Figure 3. Additional electron microscopy images of the Al_2O_3-supported samples upon reduction-oxidation-reduction treatments. a, d, g, h, i, STEM-HAADF and **b, c, e, f,** bright-field TEM of Co/Al_2O_3 at various stages of the ROR treatment. **a, b, c,** Samples after reduction at 350 °C and passivation (R-Al_2O_3); **d, e, f,** samples after reduction at 350 °C and oxidation at 200 °C (RO200-Al_2O_3); **g, h, i,** samples after reduction at 350 °C, oxidation at 200 °C and reduction at 220 °C (RO200R-Al_2O_3) and passivation.

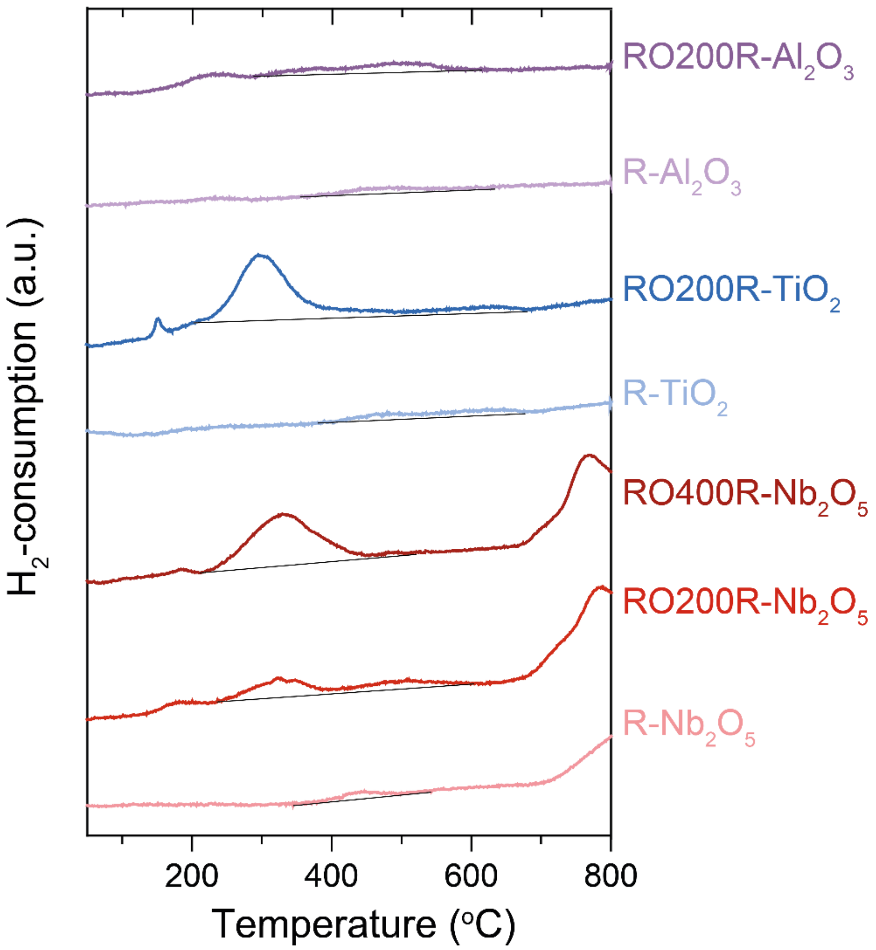

Supplementary Figure 4. TPR profiles used to determine the degree of reduction of cobalt. The baseline is shown for the peaks that were integrated as cobalt that had not been reduced and thus lowered the degree of reduction. The degree of reduction was determined after reduction at 350 °C (R-samples), after reduction at 350 °C, oxidation at 200 °C and reduction at 220 °C (RO200R-samples) and after reduction at 350 °C, oxidation at 400 °C and reduction at 220 °C (RO400R-Nb_2O_5).

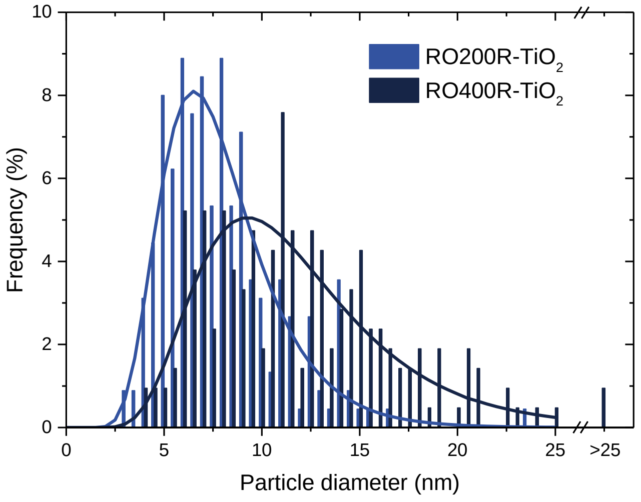

Supplementary Figure 5. Particle growth at higher oxidation temperature. Histogram of particle sizes of RO200R-TiO_2 and RO400R-TiO_2. The samples were reduced under hydrogen flow at 220 °C and then exposed to air at room temperature. Afterwards, the samples were analyzed by STEM-EDX mapping.

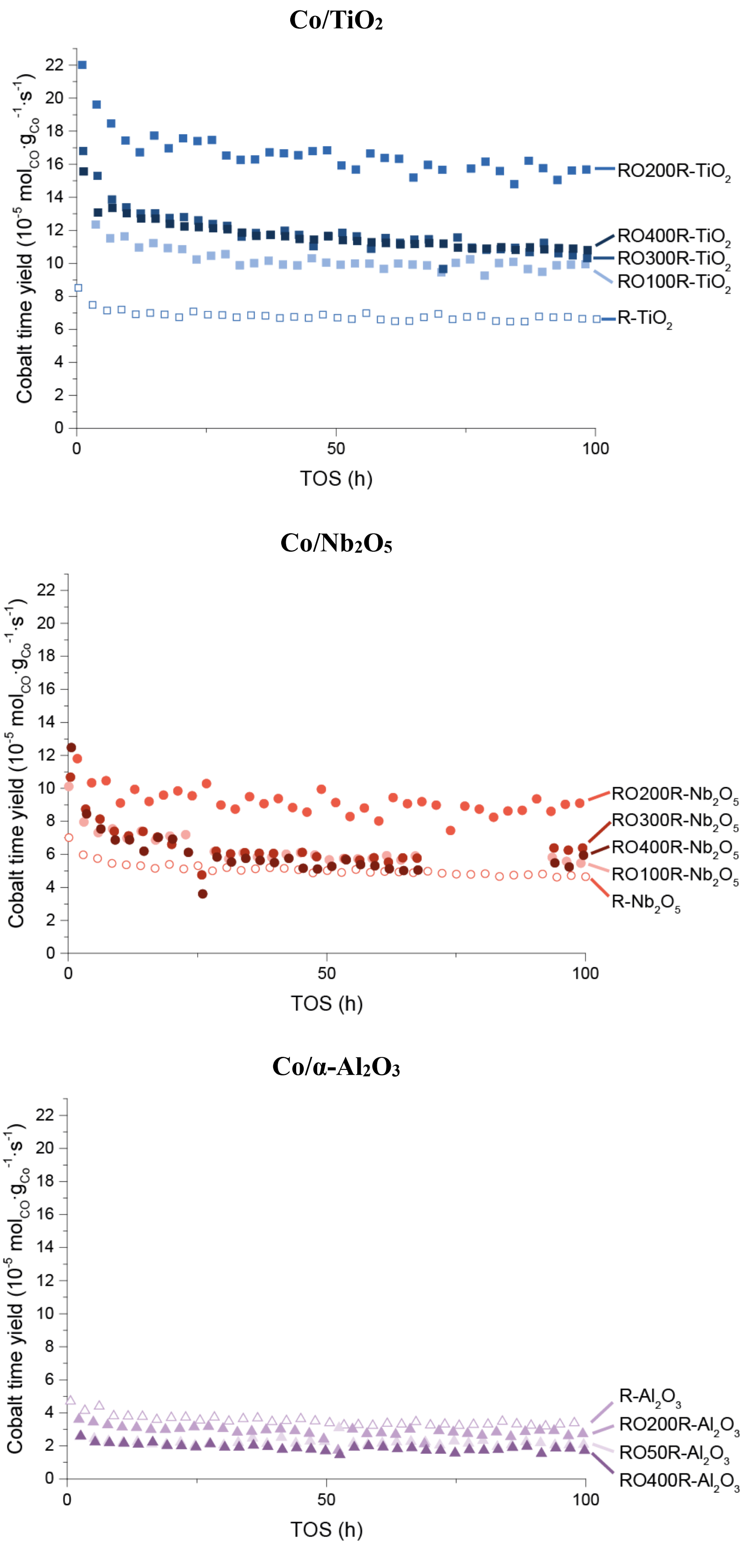

Supplementary Figure 6. Cobalt-weight-based activity over time-on-stream. Cobalt-weight-based catalytic activity of Co/TiO_2, Co/Nb_2O_5 and Co/Al_2O_3 after various reduction-oxidation-reduction treatments plotted against time-on-stream. Reaction conditions: 20 bar, 220 °C, $H_2/CO = 2$ *V*/*V*, GHSV = 1000-13000 h^{-1} and CO conversion = 15-34 % (TiO_2), 17-25 % (Nb_2O_5), 8-12 % (Al_2O_3).

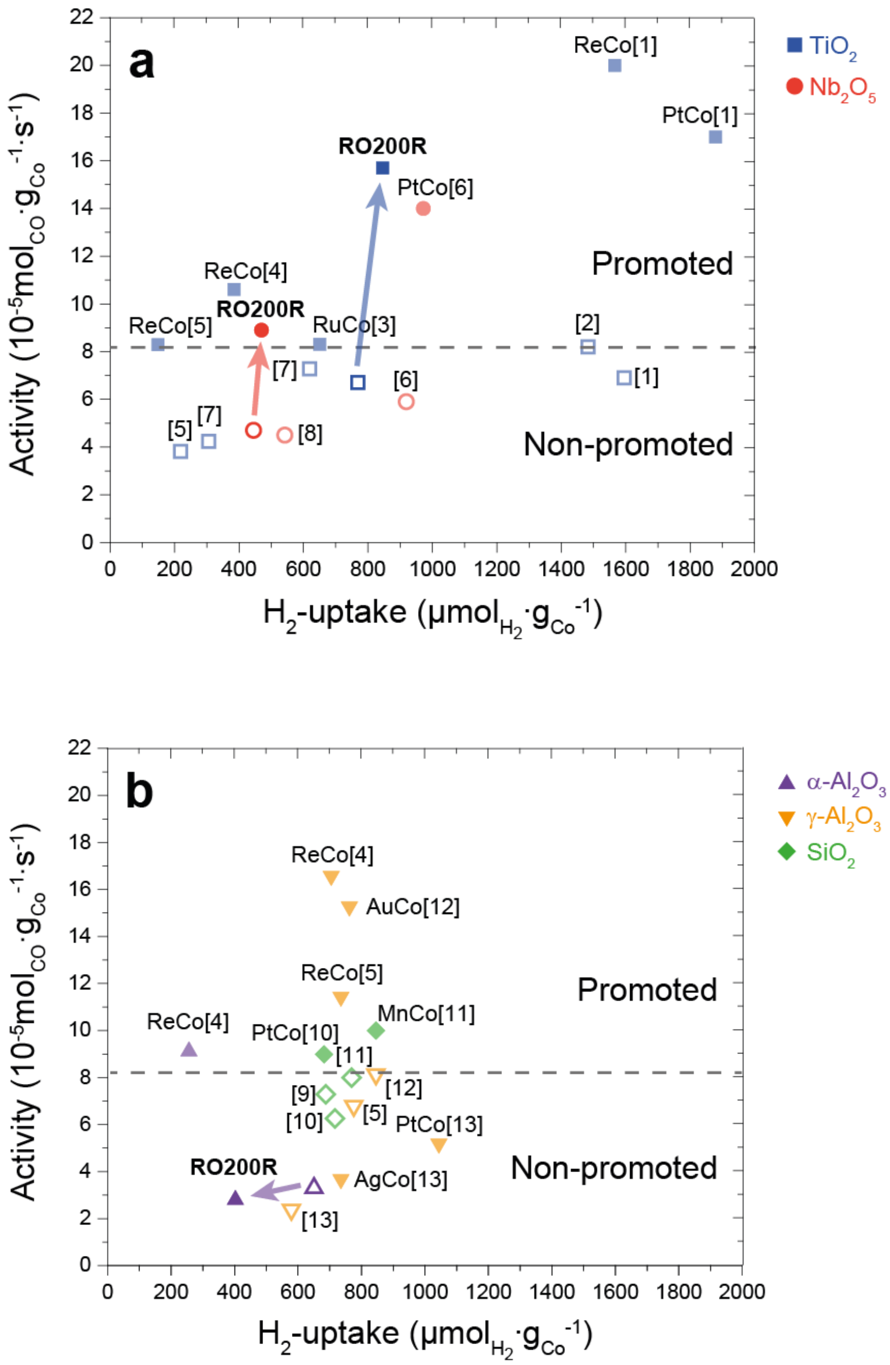

Supplementary Figure 7. Literature overview of cobalt-weight-based activities as a function of H_2-uptake. Values obtained from literature were divided into two plots: **a** shows cobalt-based catalysts supported on reducible oxides (TiO_2 and Nb_2O_5) and **b** shows cobalt-based catalysts supported on irreducible oxides (α-Al_2O_3, γ-Al_2O_3 and SiO_2). Open symbols refer to non-promoted, pristine cobalt catalysts and solid symbols to cobalt catalysts with enhanced activity either via noble metal/Mn promotion or via an ROR treatment (this work). H_2-uptake values were calculated based on the reported particle sizes from either H_2-chemisorption (refs. [7,13]), TEM or XRD. Activity values were recalculated to match the units reported here. FT was performed at 20 bar and $H_2/CO = 2$ *V*/*V* in all cases, except ref. [9] where $H_2/CO = 1$ *V*/*V*. When the catalytic performance was reported at a temperature different than 220 °C, the activity was recalculated to that at 220 °C using the Arrhenius equation with an apparent activation energy of 100 kJ·mol^{-1}.

Supplementary Tables

Supplementary Table 1. Cobalt loading of Co/TiO_2, Co/Nb_2O_5 and Co/α-Al_2O_3, as determined by inductively coupled plasma-optical emission spectroscopy (ICP-OES).

**Sample**	**Cobalt loading (wt.%)**
Co/TiO_2	6.8
Co/Nb_2O_5	5.7
Co/α-Al_2O_3	6.0

Supplementary Table 2. The degree of cobalt reduction at various stages of the ROR treatment, as measured by TPR.

**Sample**	**DOR (%)**		
	**R**	**RO200R**	**RO400R**
Co/TiO_2	97	86	n.d.
Co/Nb_2O_5	98	93	84
Co/Al_2O_3	98	95	n.d.

The degree of reduction was determined after reduction at 350 °C (R-samples), after reduction at 350 °C, oxidation at 200 °C and reduction at 220 °C (RO200R-samples) and after reduction at 350 °C, oxidation at 400 °C and reduction at 220 °C (RO400R-Nb_2O_5).

Supplementary Table 3. Summary of the catalytic performance.

Sample	GHSV (h^{-1})	CO conv. (%)	Cobalt-Time-Yield[a]	TOF (s^{-1})	Selectivity (%)		
					C_1	C_{2-4}	C_{5+}
TiO_2							
R-TiO_2	5560	20.6	6.7	0.10	8.5	7.5	84.0
RO100R-TiO_2	8290	18.2	9.8	0.11	7.0	5.0	88.0
RO200R-TiO_2	13540	19.3	15.7	0.10	7.5	4.5	88.0
RO300R-TiO_2	13540	15.1	10.5	0.074	8.8	5.4	85.8
RO400R-TiO_2	5640	34.0	10.8	0.11	6.8	4.7	88.5
Nb_2O_5							
R-Nb_2O_5	3266	17.0	4.7	0.087	8.6	6.1	85.3
RO100R-Nb_2O_5	3024	19.6	5.6	0.051	7.8	3.9	88.3
RO200R-Nb_2O_5	3874	24.6	8.9	0.068	6.7	4.2	89.1
RO300R-Nb_2O_5	3070	25.5	6.2	0.058	8.5	3.6	87.9
RO400R-Nb_2O_5	2954	19.3	5.5	0.077	11.4	4.6	85.0
α-Al_2O_3							
R-Al_2O_3	1249	12.5	3.3	0.025	10.9	14.2	85.8
RO50R-Al_2O_3	940	9.62	2.0	0.031	5.8	4.4	89.8
RO200R-Al_2O_3	952	8.98	2.8	0.035	6.2	4.1	89.7
RO400R-Al_2O_3	956	8.17	1.7	0.029	7.6	6.0	86.4

[a] in 10^{-5} $mol_{CO} \cdot g_{Co}^{-1} \cdot s^{-1}$

Reaction conditions: 20 bar, 220 °C and H_2/CO = 2 *V*/*V*. The reported data was averaged over three data points between 90-100 h on stream.

Supplementary Table 4. Lewis acidity of various metal oxides.

**Formal oxidation state of the cation (Nm)**	**Formula of the corresponding metal oxide**	**Lewis acid character (N_M-2δ_M)[a]**
5^+	Nb_2O_5	3.895
4^+	TiO_2	3.046
	NbO_2	2.877
3^+	Ti_2O_3	1.930
	Nb_2O_3	1.883
	Al_2O_3	2.274

[a] δ_M corresponds to the Sanderson's partial charge of the cation. Data was obtained from ref [14].

Supplementary Methods

Definitions of catalytic activity and selectivity

The activity of the catalysts was expressed as CO conversion, cobalt time yield (CTY) and turnover frequency (TOF). The CO conversion was defined according to (Supplementary Equation 1), CTY according to (Supplementary Equation 2) and TOF according to (Supplementary Equation 3).

$$X_{CO} = (F_{CO,in} - F_{CO,out}) \cdot F_{CO,in}^{-1} \qquad \text{(Supplementary Equation 1)}$$

$$CTY = F_{CO,in} \cdot X_{CO} \cdot V_m^{-1} \cdot m_{Co}^{-1} \qquad \text{(Supplementary Equation 2)}$$

$$TOF = CTY \cdot N_A \cdot d_{Co} \cdot SA_{Co}^{-1} \qquad \text{(Supplementary Equation 3)}$$

In these equations, X_{CO} is the CO conversion, $F_{CO,in}$ is the flow of CO into the reactor and $F_{CO,out}$ is the flow of CO in the product stream. V_m denotes the molar volume, m_{Co} the mass of cobalt in the reactor, N_A is the Avogadro constant, d_{Co} the cross-sectional area of a cobalt atom (0.0662 nm^2 according to ref. [15]) and SA_{Co} is the cobalt specific surface area as obtained by H_2-chemisorption.

The selectivity towards light hydrocarbon products with carbon number n (with $1 \leq n \leq 4$) was calculated as in (Supplementary Equation 4) and the selectivity towards the heavier fraction ($n \geq 5$) as in (Supplementary Equation 5)

$$S_{C1\text{-}C4} = F_{Cn} \cdot n \cdot (F_{CO,in} \cdot X_{CO})^{-1} \qquad \text{(Supplementary Equation 4)}$$

$$S_{C5+} = 1 - S_{C1\text{-}C4} \qquad \text{(Supplementary Equation 5)}$$

In this case, S_{Cn} represents the selectivity towards hydrocarbon product of a specific carbon number and F_{Cn} is the flow of the corresponding hydrocarbon product.

Supplementary References

[1] Eschemann, T. O., Oenema, J. & de Jong, K. P. Effects of noble metal promotion for Co/TiO_2 Fischer-Tropsch catalysts. *Catal. Today* **261,** 60–66 (2016).

[2] Eschemann, T. O. & de Jong, K. P. Deactivation Behavior of Co/TiO_2 Catalysts during Fischer–Tropsch Synthesis. *ACS Catal.* **5,** 3181–3188 (2015).

[3] Prieto, G. *et al.* Cobalt-Catalyzed Fischer-Tropsch Synthesis: Chemical Nature of the Oxide Support as a Performance Descriptor. *ACS Catal.* **5,** 3323–3335 (2015).

[4] Lögdberg, S. *et al.* Effect of water on the space-time yield of different supported cobalt catalysts during Fischer-Tropsch synthesis. *Appl. Catal. A Gen.* **393,** 109–121 (2011).

[5] Storsæter, S., Borg, Ø., Blekkan, E. A. & Holmen, A. Study of the effect of water on Fischer-Tropsch synthesis over supported cobalt catalysts. *J. Catal.* **231,** 405–419 (2005).

[6] den Otter, J. H., Yoshida, H., Ledesma, C., Chen, D. & de Jong, K. P. On the superior activity and selectivity of PtCo/Nb_2O_5 Fischer Tropsch catalysts. *J. Catal.* **340,** 270–275 (2016).

[7] Iglesia, E. Design, synthesis, and use of cobalt-based Fischer-Tropsch synthesis catalysts. *Appl. Catal. A Gen.* **161,** 59–78 (1997).

[8] Hernández Mejía, C., den Otter, J. H., Weber, J. L. & de Jong, K. P. Crystalline niobia with tailored porosity as support for cobalt catalysts for the Fischer–Tropsch synthesis. *Appl. Catal. A Gen.* **548,** 143–149 (2017).

[9] Sun, X. *et al.* Manufacture of highly loaded silica-supported cobalt Fischer–Tropsch catalysts from a metal organic framework. *Nat. Commun.* **8,** 1680 (2017).

[10] Cheng, K. *et al.* The role of carbon pre-coating for the synthesis of highly efficient cobalt catalysts for Fischer–Tropsch synthesis. *J. Catal.* **337,** 260–271 (2016).

[11] Johnson, G. R., Werner, S. & Bell, A. T. An Investigation into the Effects of Mn Promotion on the Activity and Selectivity of Co/SiO_2 for Fischer–Tropsch Synthesis: Evidence for Enhanced CO Adsorption and Dissociation. *ACS Catal.* **5,** 5888–5903 (2015).

[12] Nabaho, D., Niemantsverdriet, J. W., Claeys, M. & Van Steen, E. Hydrogen spillover in the Fischer–Tropsch synthesis: An analysis of gold as a promoter for cobalt–alumina catalysts. *Catal. Today* **275,** 27–34 (2016).

[13] Jermwongratanachai, T. *et al.* Fischer-Tropsch synthesis: Comparisons between Pt and Ag promoted Co/Al_2O_3 catalysts for reducibility, local atomic structure, catalytic activity, and oxidation-reduction (OR) cycles. *Appl. Catal. A Gen.* **464–465,** 165–180 (2013).

[14] Jeong, N. C. *et al.* Acidity Scale for Metal Oxides and Sanderson's Electronegativities of Lanthanide Elements. *Angew. Chemie Int. Ed.* **47,** 10128 –10132 (2008).

[15] Reuel, R. C. & Bartholomew, C. H. Effects of support and dispersion on the CO hydrogenation activity/selectivity properties of cobalt. *J. Catal.* **85,** 78–88 (1984).
